# Feasibility and acceptability of hypnosis-derived communication administered by trained nurses to improve patient well-being during outpatient chemotherapy: a pilot-controlled trial

**DOI:** 10.1007/s00520-021-06481-6

**Published:** 2021-08-10

**Authors:** Caroline Arbour, Marjorie Tremblay, David Ogez, Chloé Martineau-Lessard, Gilles Lavigne, Pierre Rainville

**Affiliations:** 1 Hôpital du Sacré-Cœur de Montréal, CIUSSS du Nord-de-L’Île-de-Montréal, 5400 Boul. Gouin Ouest, Room: E-1381, Montreal, QC H4J 1C5 Canada; 2 Faculty of Nursing, Université de Montréal, Montreal, QC Canada; 3 Hôpital de La Cité-de-La-Santé, CISSS de Laval, Laval, QC Canada; 4 Faculty of Medicine, Université de Montréal, Montreal, QC Canada; 5 Centre de Recherche de L’Hôpital Maisonneuve-Rosemont, Montréal, Québec Canada; 6 Faculty of Dental Medicine, Université de Montréal, Montreal, QC Canada; 7 Institut Universitaire de Gériatrie de Montréal, CIUSSS du Centre-Sud-de-L’Île-de-Montréal, Montreal, QC Canada

**Keywords:** Cancer, Chemotherapy, Hypnosis, Emotional support, Symptom management

## Abstract

**Purpose:**

This pilot-controlled trial aimed to examine the feasibility and acceptability of hypnosis-derived communication (HC) administered by trained nurses during outpatient chemotherapy to optimize symptom management and emotional support — two important aspects of patient well-being in oncology.

**Methods:**

The trial was conducted in two outpatient oncology units: (1) intervention site (usual care with HC), and (2) control site (usual care). Nurses at the intervention site were invited to take part in an 8-h training in HC. Participants’ self-ratings of symptoms and emotional support were gathered at predetermined time points during three consecutive outpatient visits using the Edmonton Symptom Assessment Scale and the Emotional Support Scale.

**Results:**

Forty-nine patients (24 in the intervention group, 25 in the control group) with different cancer types/stages were recruited over a period of 3 weeks and completed the study. All nurses (*N* = 10) at the intervention site volunteered to complete the training and were able to include HC into their chemotherapy protocols (about ± 5 min/intervention). Compared to usual care, patients exposed to HC showed a significant reduction in physical symptoms during chemotherapy. In contrast, perception of emotional support did not show any significant effect of the intervention. Participants exposed to HC report that the intervention helped them relax and connect on a more personal level with the nurse during chemotherapy infusion.

**Conclusions:**

Our results suggest that HC is feasible, acceptable, and beneficial for symptom management during outpatient chemotherapy. While future studies are needed, hypnosis techniques could facilitate meaningful contacts between cancer patients and clinicians in oncology.

**Trial registration:**

Clinical Trial Identifier: NCT04173195, first posted on November 19, 2019

**Supplementary Information:**

The online version contains supplementary material available at 10.1007/s00520-021-06481-6.

## Introduction

The number of cancer cases requiring chemotherapy is on the rise around the world, and most treatments are administered by nurses in an outpatient oncology unit [1]. Constant advances in cancer treatments have led to great improvements in patient survival rates [2, 3]. However, cancer treatments, including chemotherapy, often bring unwanted symptoms or side effects such as nausea, fatigue, and anxiety [4]. Accordingly, management of these symptoms and psychological distress is considered a core feature of nursing practice in oncology. Despite the fact that patient satisfaction is generally high in outpatient cancer [5–8], one-third of patients report that healthcare professionals are not doing everything in their power to optimize symptoms management and emotional support during chemotherapy [9]. On the other hand, attending to the individual patients needs can be a challenge in time-constrained oncology units [10, 11].

Hypnosis-derived communication (HC) has emerged as a promising approach to promote pain relief and patient well-being during cancer treatments such as radiation therapy and cancer surgery [12, 13]. HC is a simple form of conversation which integrates the language patterns of clinical hypnosis such as verbal repetition, suggestion, and metaphor. One of the strategies commonly employed in HC is to redirect one’s attention from the source of stress to more relaxing mental images, thus reducing alertness to unpleasant feelings and thoughts. Although patients do not have to be explicitly aware that they are being subjected to HC to experience the therapeutic benefits (a phenomenon referred to covert hypnosis), several models stress the importance of the partnership between patients and clinicians [14]. Providing choices and taking into account patients’ concerns and preferences is also a fundamental principle of HC [15].

A large body of evidence supports the feasibility and the soothing properties of HC during various medical procedures [12, 13, 16]. Research in a specific cancer population has also supported the use of HC for persistent symptoms management after cancer treatments [17–19]. While the potential benefits of HC for cancer-related symptoms are evident, practical barriers can diminish implementation of such interventions in outpatient oncology units. These barriers include not only limited resources, but also time constraints since nurses can rarely focus on one patient for a sustained period of time. To address such gaps, a brief HC intervention was adapted and translated from a previously published protocol [13], with the objective that the typical oncology nurse would be able to successfully integrate HC into his/her practice surrounding chemotherapy treatments.

## Study aims

The primary aims of the pilot trial were to examine the feasibility and acceptability of HC administered by trained nurses working in an outpatient clinic. The secondary aim was to test whether such intervention can be successfully used by nurses to improve symptom management and perceived emotional support during outpatient chemotherapy. The study followed the CONSORT recommendations for pilot and feasibility studies [20] (see Supplementary information).

## Methods

### Study design and sites

The pilot trial was conducted simultaneously in two outpatient oncology units from the same metropolitan area. Both units also share several oncologists, ensuring a certain uniformity in the delivery of chemotherapy and symptom management protocols between sites. To prevent “contamination” between groups, site 1 served as the intervention site (combining usual care with HC during chemotherapy) and site 2 served as the control site (providing usual care only during chemotherapy). In both sites, chemotherapy treatments were administered in an open room attended by multiple patients at the same time. Curtains were used to divide the space between each chair, which is reclining so patients could lie back to rest during treatment. Rolling stools were available for nurses to sit comfortably beside patients for catheter installation and other care.

### Compliance with ethical standards

The study was conducted in accordance with the Helsinki Declaration and approval was granted from the Institution Review Board (2019–1751). All participants provided written consent.

### Eligibility

Eligibility requirements were the same at both sites. Briefly, patients 18 years or older, with a cancer diagnosis (any type or stage) for less than 2 years, having initiated a cycle of chemotherapy since at least 1 week, expected to receive at least two additional cycles related to this treatment, and not participating in any other research protocol were considered eligible. Patients non-fluent in French, with a hearing impairment, as well as those with a pre-cancer history of chronic pain or major mental health difficulty were excluded. Patients ≥ 65 years were screened for the presence of mild cognitive impairments before recruitment and excluded when Mini-Mental State score was ≥ 21 [21].

### Recruitment

Eligible patients were met by the research assistant (RA) assigned to each site during a routine visit to the outpatient unit. To avoid study bias, participants were not made aware at the time of recruitment that the nurse-led intervention would be derived from the principles of clinical hypnosis. Rather, they were told that the project aimed to explore the effects of different nursing communication approaches on patients’ level of general well-being during chemotherapy. Reasons for incomplete disclosure and group assignment were revealed to participants from the intervention group by the RA during a debriefing session at the end of the study.

### Procedure

Data collection took place during three consecutive visits to the outpatient oncology unit, spaced approximately 2 weeks apart. During these visits, information about participants’ cancer-related symptomatology and their perception of emotional support was gathered at specific time points by the RA (see illustration of the study procedure in Supplementary information). At no time during the study protocol, nurses in charge of HC intervention were made aware of this information.

Specifically, visit 1 was planned for recruitment and baseline characterization (T0) using a short sociodemographic form and self-reported questionnaires about cancer symptomatology and emotional support. Visit 2 was reserved for the deployment of HC at the beginning of chemotherapy in participants from the intervention group (site 1), while participants from the control group (site 2) received usual care. During this visit, the same self-reported questionnaires were administered before (T1) and immediately after (T2) chemotherapy. In addition, nurses in charge of administering HC were asked about the presence of any adverse event or reaction during the intervention. Visit 3 marked the end of the research protocol. As part of this last visit, the self-reported questionnaires were completed again (T3) upon participants’ arrival at the outpatient unit, to assess potential carry-over benefits across visits. After debriefing, participants from the intervention group were asked to provide their general appreciation of HC.

### Intervention

The original script used for the HC intervention was validated in four randomized clinical trials with *N* = 738 outpatients [12, 13, 22, 23]. The script was translated in French using the forward–backward translation method (CA, CML) and revised by two experts in clinical hypnosis (MT, DO) for final approval. The final script was separated into three complementary parts where the nurse invites the patient to (1) focus on respiration and picture him/herself floating over a pleasant setting of his/her choice, (2) transform any potential discomfort into a more tolerable sensation of his/her choice, and (3) project his/her concerns onto an imaginary screen and find solutions (full version of the script provided in Supplementary information). The script, consisting less than two pages of text, could easily be memorized by nurses, although a pocket-sized memory aid was provided to them. To ensure a minimum of uniformity, nurses were advised to start the HC intervention within 5 min of the initiation of chemotherapy treatment. Furthermore, nurses were instructed to pull the curtain around the patients to promote the sensation of intimacy during the intervention.

### Nurses training

Staff nurses working at the intervention site were invited to take part, on a voluntary basis, to a standardized training in HC over an entire day (from 8 AM to 4 PM). Training was remunerated and carefully planned to fall between visit 1 and visit 2 of the study protocol to ensure that baseline values in the intervention site would not be contaminated. The content of the training was developed and led by two members of the research team (MT, DO) based on a previously validated training [16]. The first part of the session addressed general principles of hypnosis, the correct use of suggestion, the importance of the control perception, relaxation training, and hypnotic language. Using excerpts from lectures, demonstrations, and supervised exercises, the second part of the session focused on practicing some HC principles and the actual script provided for the study. At the end of the training, a manual with the teaching content, examples, and the script in a detachable form was provided to the nurses. One member of the research team (MT) provided on-site support during the first days of the study to assist nurses in the smooth running of the HC intervention.

### Measures

#### Feasibility and acceptability

Feasibility was measured in terms of participants’ recruitment and retention. Recruitment was defined as the number of patients enrolled, considering the number of patients that were approached for participation. Reasons for participation refusal were recorded. Retention data included information on the number of participants who were enrolled at visit 1 and completed all parts of the study (visits 2 and 3). Acceptability of the HC intervention was defined by nurses’ training attendance rate, ease of HC integration into routine chemotherapy protocols, and reports of adverse event or reaction during the HC intervention.

#### Sociodemographic and cancer characteristics

Sociodemographic information (i.e., age, gender, ethnic background, education, marital status, current employment status) as well as information about cancer type was gathered at visit 1 for sample description.

#### Self-reported questionnaires

The French versions of the Edmonton Symptom Assessment Scale (ESAS) and the Emotional Support Scale (ESS) were used to gather information about participants’ overall well-being [24, 25]. The ESAS consists of nine self-reported items that evaluate a mix of six physical symptoms (i.e., pain, fatigue, drowsiness, nausea, appetite, shortness of breath) and two psychological symptoms (i.e., depression, anxiety), in addition to a global sense of discomfort. Each scale is graded from 0 to 10 (0 indicating “no symptom or discomfort” and 10 “worst possible symptom or discomfort”). The ESAS has good internal validity (α Cronbach = 0.79) and excellent test–retest reliability (Spearman’s *r* = 0.86) in oncology [26]. To measure perceived emotional support, we adopted the 16-item ESS that was originally developed in the context of family and student–teacher relationship [27]. This scale assesses the perception of receiving encouragement, compassion, and other forms of emotional support from close others [25]. Of the 16 initial items, four items were removed as they were not representative of the type of support that is expected between a patient and a clinician (such as advice against bullying or expression of love). For the 12 remaining statements, participants were asked to think about the most recent encounter they had with a nurse during chemotherapy and then to indicate the extent to which they were satisfied with the demonstration of emotional support on a 100-mm visual analogue scale (0 indicating “total absence of satisfaction” and 100 “maximal satisfaction”). Prior abbreviated versions of the ESS showed excellent internal validity (α Cronbach > 0.90) [28].

#### Analysis

Descriptive statistics (frequencies with percentages or means with standard deviations) were calculated on all variables associated with feasibility (recruitment, retention), acceptability (training attendance, ease of use, adverse events), and baseline characteristics (at both the sample level and group level). Considering this study was a pilot, and there was no prior data to support adequate power calculation, statistical group comparisons were kept to a minimum. Thus, three composite scores were derived from the ESAS according to a previously proposed method [29], leading to a physical subscore (total of six physical symptoms, score range 0–60), a psychological subscore (total of 2 psychological symptoms, score range 0–20), and a global discomfort score (physical score + psychological score + discomfort item, score range 0–90). The ESS total score was used as the sole measure for perceived emotional support. Considering questionnaire data gathered at visit 1 (baseline) and visit 3 (debriefing) were normally distributed, RMANOVA was used to compare groups against the ESAS and ESS evaluations obtained at different measurement times, followed by post hoc comparisons test in case of significant results. Since questionnaire data gathered at visit 2 were not always normally distributed for post-intervention data, median changes in questionnaire scores from pre-intervention to post-intervention were computed, and groups were compared by means of Mann–Whitney *U* tests. Effect sizes were calculated using Cohen’s partial *η*2 values [30]. Values of 0.01, 0.06, and 0.14 were considered as small, medium, and large effect sizes, respectively. All tests were two-tailed and results were considered to be significant at *P* < 0.05.

## Results

### Feasibility

**Fig. 1:**
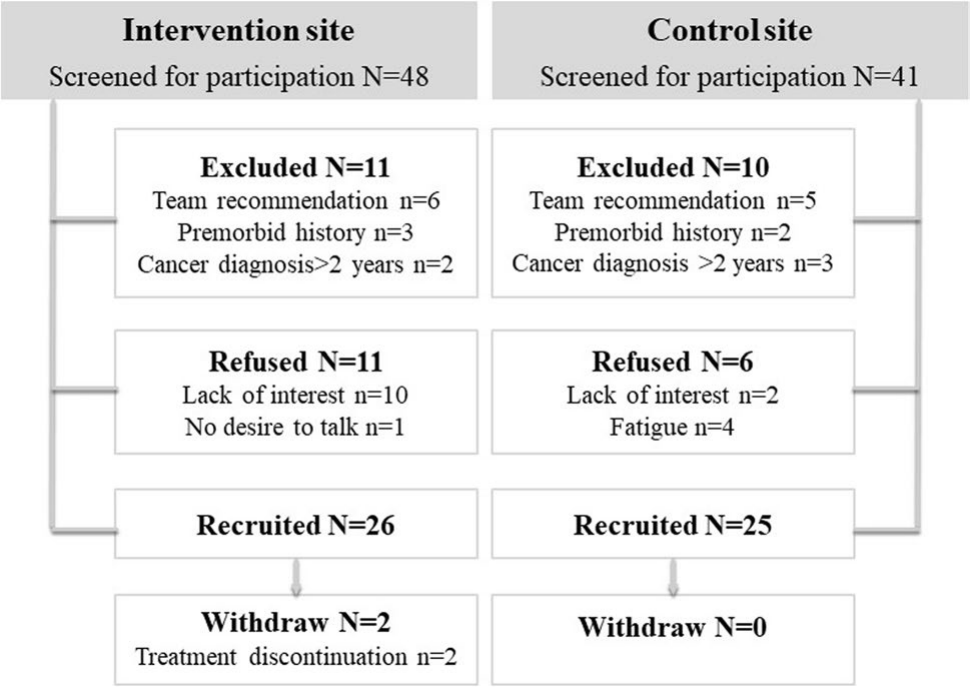
Study screening and recruitment flow diagram

Eighty-nine eligible patients were screened over a period of 3 weeks in November 2019 and 68 were approached for participation (37 at the intervention site, and 31 at the control site) (see Fig. 1). Of them, 17 refused to take part in the study (11 at intervention site, six at control site). Reasons for declining participation were lack of interest in the project, fatigue, and absence of desire to talk during treatment. Recruitment rate was 70% and 81% at the intervention site and the control site, respectively. Two participants from the intervention site had to be withdrawn from the study because of intravenous chemotherapy discontinuation, leading to a retention rate of 92% at this site. There were no dropouts or withdrawals at the control site. All remaining participants completed baseline and subsequent measures.

### Sample characteristics

The final sample consisted of 49 patients (24 in intervention group, 25 in control group). Table 1 shows their baseline characteristics. The average age was 63 years, and most were Caucasian (88%), and they were either married or living with a partner (68%). All patients had a high school education or higher. Aside from ethnicity and employment status, which showed different shape of distributions, there was no apparent between-group difference in sociodemographic and cancer characteristics. Groups were comparable at baseline on the physical and psychological subscores of the ESAS, as well as the total score of the ESAS and ESS questionnaire (all *P*s > 0.1).

**Table 1:** Participants’ sociodemographic and cancer characteristics in each group

	Total sample (*N* = 49)	Intervention group (*N* = 24)	Control group (*N* = 25)	*χ*^2^ or t test
*Age (years)*				
Mean (SD)	63 (12)	62 (12)	65 (12)	ns
Range	32–89	35–89	32–84	
*Gender, N (%)*				
Women	25 (51)	12 (50)	13 (52)	ns
Men	24 (49)	12 (50)	12 (48)	
*Marital status, N (%)*				
Married/living with partner	33 (68)	13 (54)	20 (80)	ns
Single	6 (12)	4 (17)	2 (8)	
Widowed	6 (12)	4 (17)	2 (8)	
Divorced/separated	4 (8)	3 (13)	1 (4)	
*Ethnicity, N (%)*				
Caucasian	43 (88)	18 (75)	25 (100)	*P* = 0.03
African American/black	4 (8)	4 (17)	–	
Other	2 (4)	2 (8)	–	
*Education level, N (%)*				
High school	21 (43)	10 (41)	11 (44)	ns
College degree	11 (22)	4 (17)	7 (28)	
University degree	17 (35)	10 (41)	7 (28)	
*Employment status, N (%)*				
Employed full time	8 (16)	7 (29)	1 (4)	*P* = 0.02
Employed part time	3 (6)	2 (8)	1 (4)	
Incapacity of work/invalidity	9 (18)	6 (25)	3 (12)	
Unemployed/retired/other	29 (60)	9 (38)	20 (80)	
*Cancer diagnosis, N (%)*				
Breast cancer	11 (22)	5 (21)	6 (24)	ns
Digestive cancer (colorectal, pancreas)	9 (18)	3 (12)	6 (24)	
Blood cancer (lymphoma, myeloma)	10 (21)	5 (21)	5 (20)	
Lung cancer	10 (21)	6 (25)	4 (16)	
Prostate cancer	7 (14)	4 (17)	3 (12)	
Others (bladder, thyroid)	2 (4)	1 (4)	1 (4)	

### Acceptability

Of the ten staff nurses working at the outpatient oncology unit at the intervention site, all of them voluntarily enrolled and completed the HC training. Although this was not an explicit objective of the study, nine nurses (90%) reported in the feedback and satisfaction survey that they found the training extremely relevant for their practice. In addition, six nurses (60%) reported that they were convinced that HC could truly have an impact on patient well-being at the outpatient oncology unit. After study initiation, the entire sample of trained nurses reported being able to include HC into their chemotherapy protocols. While the first few HC applications took more time, nurses reported that application of the HC script lasted on average 5 ± 2 min once they were more at ease with the intervention. Importantly, nurses reported no patient adverse event following the administration of the HC intervention.

### Impact of the intervention on patients’ cancer-related symptoms and emotional support

**Fig. 2:**
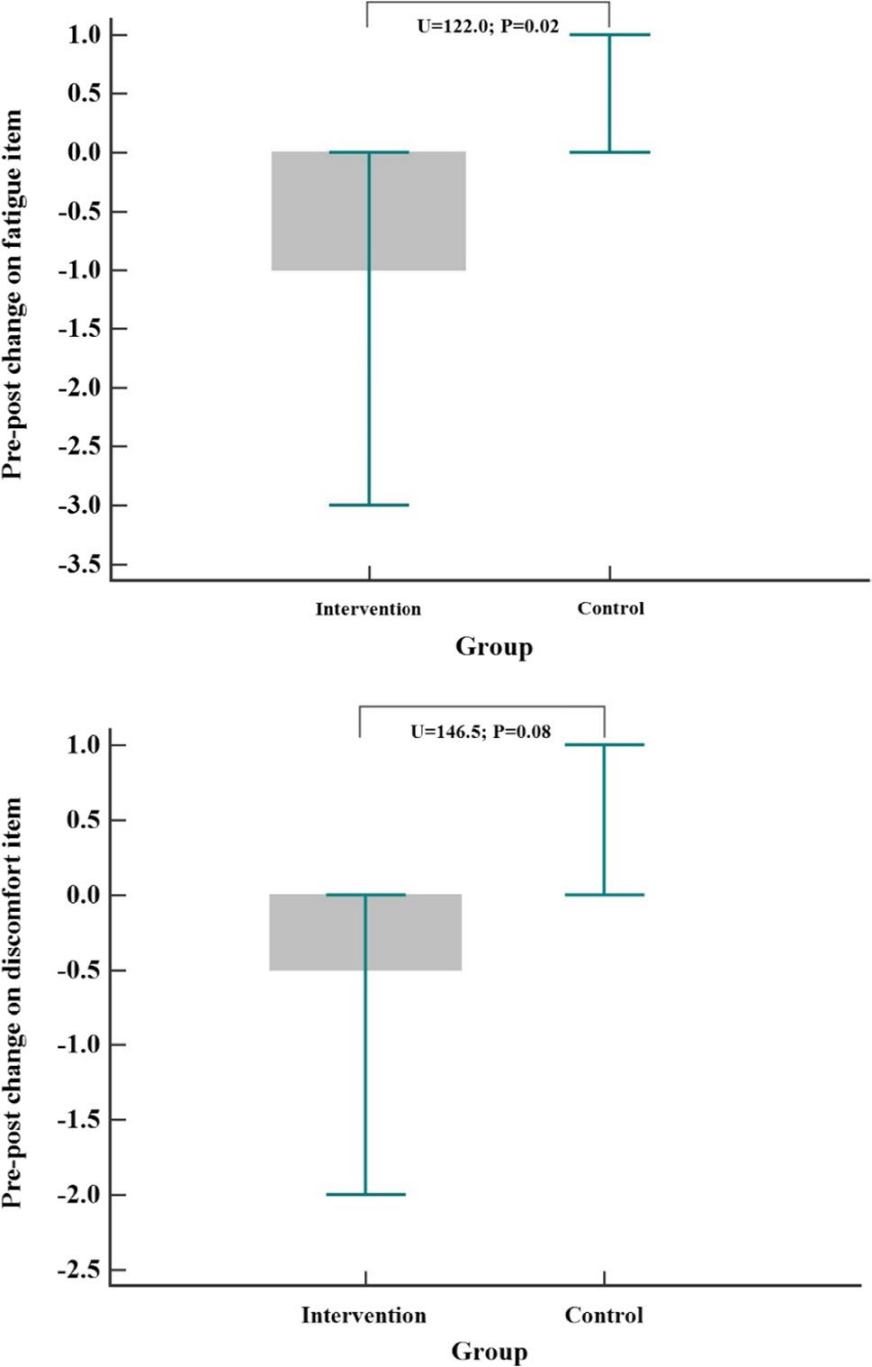
Shows exploratory and uncorrected post hoc comparison of patients’ score fluctuation from pre- to post-intervention on the fatigue and discomfort item of the ESAS questionnaire. The upper and lower whiskers represent the 75th and 25th quartiles, respectively

Considering that the data gathered at visit 2 were not normally distributed, between-group differences in cancer-related symptom evolution from T1 (pre-intervention) to T2 (post-intervention) were assessed with Mann–Whitney *U* tests (see Table 2). During chemotherapy treatment, patients in the intervention group experienced significant reductions in ESAS physical symptoms (*U* = 111.00, *P* < 0.001) and global discomfort (*U* = 106.50, *P* = 0.003) after being exposed to HC. No group difference was observed for the ESAS psychological subscore and ESS score. The partial *η*2 values for the ESAS physical symptoms subscore and global discomfort score were 0.25 and 0.21, respectively. Follow-up contrasts suggest a decrease in fatigue and discomfort in the intervention group compared to that in the control group (see Fig. 2).

**Table 2:** Description and comparison of symptoms and emotional state from pre- to post-intervention

	Intervention group (N = 24)			Control group (N = 25)			Comparison	
	T1 pre-chemotherapy	T2 post-chemotherapy	T2-T1 pre-post change	T1 pre-chemotherapy	T2 post-chemotherapy	T2-T1 pre-post change	T2-T1 group difference	Effect size
	*Median* *(IQR)*	*Median* *(IQR)*	*Median* *(IQR)*	*Median* *(IQR)*	*Median* *(IQR)*	*Median* *(IQR)*	*U* *P-value*	*Partial η*2
ESAS questionnaire								
Physical subscore (0–60)	5.9 (3.2–13.6)	4.2 (0.1–7.3)	− 2.7 (− 4.3–0.0)	3.7 (0.9–9.2)	6.6 (1.9–13.0)	0.4 (− 0.4–3.7)	111.0 *P* < 0.001	0.25
Psychological subscore (0–20)	0.9 (0.0–2.0)	0.6 (0.0–1.2)	0.0 (− 1.2–0.8)	0.5 (0.0–0.9)	0.9 (0.0–2.2)	0.0 (− 0.2–0.3)	214.5 *P* = 0.391	0.02
Global discomfort score (0–90)	10.0 (3.9–16.9)	5.3 (0.3–10.7)	− 4.1 (− 6.3–0.6)	6.0 (1.3–10.5)	8.4 (1.4–23.1)	0.2 (− 1.9–4.3)	106.5 *P* = 0.003	0.21
ESS questionnaire								
Emotional support score (0–120)	120.0 (115.1–120.0)	120.0 (114.1–120.0)	0.0 (0.0–3.2)	117.0 (113.4–119.4)	114.9 (103.1–119.5)	− 0.5 (− 3.9–0.6)	20.0 *P* = 0.662	0.02

### Carry-over effects and debriefing

No significant group effect was revealed by the RMANOVAs performed on symptomatology and emotional support data gathered during the last visit of the protocol (visit 3), suggesting the absence of carry-over effects. During debriefing, 20 participants from the experimental group (83%) reported that HC helped them relaxed during chemotherapy treatments. Fifteen participants (63%) reported that the intervention could be more beneficial for long-term symptom management as HC could be reapplied, if needed, with symptom evolution and not just during chemotherapy. Two thirds of participants (16 out of 24) also reported that HC led them to connect and experience a more personal moment with the nurse when normally it is more specific to treatments.

## Discussion

This study examined the feasibility, acceptability, and outcomes of a nurse-led HC intervention during outpatient chemotherapy. The intervention consisted of a brief and partially scripted text that could easily be memorized by nurses to help them develop their own language and skills. Intervention feasibility was supported by the successful recruitment of 51 patients in 3 weeks, of which only two had withdrawn because of chemotherapy cessation. In terms of acceptability, all staff nurses at the intervention site voluntarily completed the HC training during the study protocol. Ninety percent of them reported being very satisfied with the training. While nurses were able to easily integrate HC into their usual care surrounding chemotherapy, one challenge they faced was the appropriation of the script during the first few interventions. Future studies on the topic should consider increasing the expert coaching phase and the training of on-site facilitators to support protocol implementation and nursing HC delivery.

Although the sample size was small, a significant reduction in physical symptoms and global discomfort scores was observed in the intervention group compared to that in the control group. Exploratory analyses suggest that these reductions were more pronounced for multidimensional items such as fatigue and global discomfort. It is also encouraging to observe that while the average scores for pain, drowsiness, and nausea were found to increase in the control group following chemotherapy, they appeared to remain quite stable in the intervention group. Although the exact mechanisms by which HC specifically acts, we can cautiously extrapolate from the neuroimaging literature on clinical hypnosis. Based on these studies [31–35], the soothing properties of hypnosis depends on the activation of frontal cortical areas, particularly the dorsolateral prefrontal cortex involved in executive functions such as planning and selective attention, and the median prefrontal cortex involved in the regulation of attention and emotions. Independent of the underlying mechanisms, the majority of participants found HC to be a nice and relaxing addition for symptom management during chemotherapy. Future studies should examine the utility of such intervention not only during treatments but also as self-management strategies between them [36], as suggested by some participants.

Despite prior evidence showing that even brief hypnosis interventions can have notable effects on emotion regulation [37], the expected benefits of HC on the perception of emotional support was not observed in this study. Similarly, no significant changes in the items relating to psychological well-being (i.e., anxiety, depression) were found. While these results can easily be explained by the fact that perception of emotional support was already high in both experimental groups at baseline, we cannot rule out on the possibility that psychological distress (which is typically common in cancer patients) was also underrepresented in our study sample. Indeed, patients were recruited within the first 2 years of their cancer diagnosis, during a time frame where hope of a favorable outcome is typically high [38]. In addition, 11 patients were not approached for participation (six in the intervention site and five in the control site) following the clinical team recommendation that they were not in the best shape or state of mind to participate to a research protocol. In retrospect, these patients could have been the ones who would have most benefited from the potential emotional regulating benefits of HC. Still, even if these patients had been included in the study, it is possible that our HC intervention would have had no impact on these items as multi-dose intervention is generally required for more complex mental issues.

Several aspects should be considered when interpreting findings from this pilot study. Although HC was feasible, it was only administered once in the intervention group leaving no possibility to explore the cumulative effects on patients’ well-being. Additionally, the sample size was small, thereby limiting the power to examine HC therapeutic benefits according to patients’ cancer type or symptomatology profile. The composition of the experimental groups in terms of ethnicity and employment status was uneven. As this was a pilot, exclusion criteria were kept to a minimum leading to the possibility that some aspects of patients’ general condition may have interfered with their response to HC. Lastly, even if HC was found acceptable for recently trained nurses working in the outpatient oncology unit, more research is needed to unveil the link between training and HC transfer into practice. Despite these limitations, this study contributes to the growing literature on hypnosis-derived interventions in oncology care.

## Conclusions

To conclude, our findings suggest that HC is feasible and well-accepted by oncology nurses working at the outpatient oncology unit. This study also demonstrates that with only 8 h of HC training, nurses were able to make a difference in patients’ well-being during chemotherapy. While the brief HC intervention in this study was administered by nurses in charge of chemotherapy, it could have been administered by any trained member of the interdisciplinary team [22, 23]. Until hypnosis becomes more widely accessible, this study contributes to the practical demonstration of how oncology nurses can integrate more psychosocial care in the outpatient unit. With time constraints and limited resources oncology clinicians often face, HC could be a way to achieve soothing care in a more efficient manner.

## Supplementary Information

Below is the link to the electronic supplementary material.

Supplementary file1 (DOCX 124 KB)

Supplementary file2 (DOCX 14 KB)

Supplementary file3 (DOC 227 KB)

## Data Availability

The datasets generated during and/or analyzed during the current study are available from the corresponding author on reasonable request.

## Code availability

N/A
