# Supplementary figures and images for: Feasibility and acceptability of hypnosis-derived communication administered by trained nurses to improve patient well-being during outpatient chemotherapy: a pilot-controlled trial
Source: Support Care Cancer. 2021 Aug 10;30(1):765–73. doi: 10.1007/s00520-021-06481-6 (PMC8636401; doi:10.1007/s00520-021-06481-6)

**SUPPLEMENTARY MATERIAL**

**Study procedure**

**
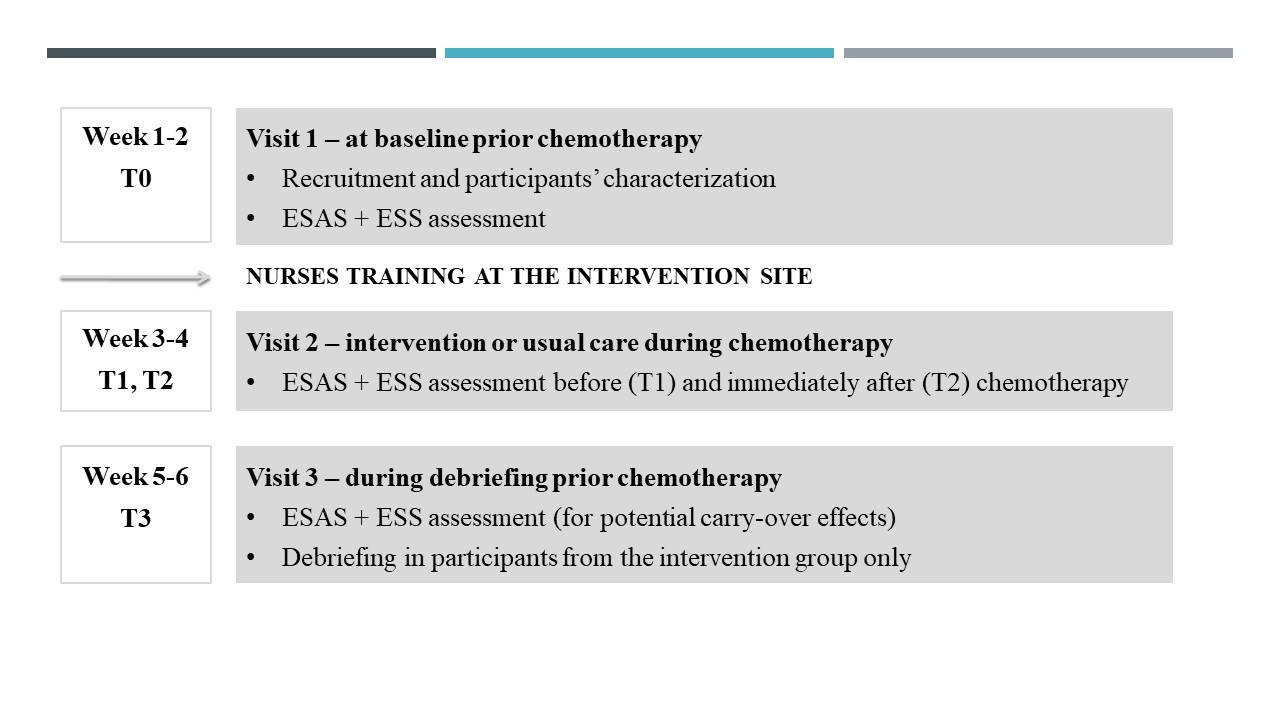
**

Supplement: Supplementary file 1 — Supplementary file1 (DOCX 124 KB) [file 520_2021_6481_MOESM1_ESM.docx]
