# Supplementary material for: Feasibility and acceptability of hypnosis-derived communication administered by trained nurses to improve patient well-being during outpatient chemotherapy: a pilot-controlled trial
Source: Support Care Cancer. 2021 Aug 10;30(1):765–73. doi: 10.1007/s00520-021-06481-6 (PMC8636401; doi:10.1007/s00520-021-06481-6)
Supplement: Supplementary file 2 — Supplementary file2 (DOCX 14 KB) [file 520_2021_6481_MOESM2_ESM.docx]

**SUPPLEMENTARY MATERIAL**

**Full version of the script** **(adaptation from Lang et al., 2006)**

I would like to suggest a concentration exercise today. Much like watching a movie or reading a captivating book. It may help make the chemotherapy treatment more comfortable. Would you like to try?

*Wait for the patient to give permission to start. Otherwise, document his/her reasons for refusing to participate and return to usual care.*

Let's start. I will show you how you can use your imagination to enter a state of physical relaxation. If you hear any sounds or noises around you, just use them to deepen your experience. Let yourself be guided by my voice. Above all, only use the suggestions that are useful to you.

On one, slowly close your eyes and take a deep breath.

On two, exhale deeply (*say at the beginning of patient exhalation*).

*Wait until the patient has finished exhaling before pursuing.*

Now imagine your whole body floating, floating through the chair you are sitting on, each breath deeper and easier. Right now, I want you to imagine that you are floating in a bath, a lake, a spa or just in space, somewhere safe and comfortable, each breath deeper and easier. With each breath, you release a little more tension from your body leaving your whole body to float, safely and comfortably, with each breath getting deeper and easier.

While staying in this state of focus, describe to me how your body is feeling right now. Where are you now? Can you imagine what it looks like? Can you smell the air? Can you see what is around you? Well, now this is your safe and pleasant place and you can use it in a way. Your body should be here, but not you. So spend your time being in the place you prefer to be.

*Wait a few seconds for the patient to soak up the sensation before pursuing.*

Now, with your eyes closed and in this state of focus, describe how you are feeling right now.

*(1) If the patient reports being in a safe and comfortable place, reinforce this feeling.*

Good. You are doing well. Keep going.

*(2) If the patient reports experiencing any form of discomfort or emotional distress.*

The (*insert the symptom or emotion*) is there, but see if you can act upon it (*by adding heat, cold, or make it lighter or heavier depending on the symptom or sensation described*)? With each breath, you release a little more tension from your body. Your whole body to float. Breath deeply.

*(2.1) If the patient is starting to feel relieved.*

Good. Keep focusing on what you are doing to get this soothing sensation.

*(2.2) If the patient is still reporting pain or another symptom.*

Try to focus on the sensations taking place in another part of your body. Now rub your fingertips together and notice all the delicate sensations in your fingers and see how long you can stay focused on that sensation. With your eyes still closed, describe to me your safe and comfortable place and the soothing sensation it generates in you. (*Wait for the patient to describe*). How do you feel now?

*End the intervention*

Thank you for agreeing to take part in this exercise with me. Know that no matter what happens during chemotherapy treatment, there is always something you can do to improve your comfort. If you enjoyed the effect it had on you, you can continue to focus on feeling your body floating, comfortable and secure.
